# Assessment of the quality of patient-oriented information over internet on testicular cancer

**DOI:** 10.1186/s12885-018-4436-0

**Published:** 2018-05-02

**Authors:** Anton S. Prasanth, Umesh Jayarajah, Ranganathan Mohanappirian, Sanjeewa A. Seneviratne

**Affiliations:** 1 Faculty of Medicine, University of Colombo, Colombo 8, Sri Lanka; 2 Department of Surgery, Faculty of Medicine, University of Colombo, P.O. Box 271, Kynsey Road, Colombo 8, Western Province Sri Lanka

**Keywords:** Quality, Testicular cancer, Information, Patient education websites

## Abstract

**Background:**

This study aimed to assess the quality and readability of patient education information available on the internet on testicular cancer.

**Methods:**

Internet searches were performed using the keywords ‘testicular cancer’, ‘testicular tumour’, ‘testicular tumor’, ‘testicular malignancy’, ‘germ cell tumour’ and ‘germ cell tumor’ using Google, Yahoo! And Bing search engines with default settings. The first 50 web links appeared in each search engine were evaluated for their readability by using the validated Flesch Reading Ease Score (FRES) while accessibility, usability and reliability were assessed using the LIDA tool. The quality was assessed using DISCERN instrument. Non-parametric tests were used for statistical analysis.

**Results:**

Overall, 900 websites were assessed and 62 websites were included in the analysis. Twenty two (22) websites (35.5%) were certified by Health on the Net Foundation code of conduct (HON code). The majority (*n* = 57, 91.9%) were non-governmental websites. The median FRES score was 51.6 (range: 28.1–74.1), the overall median LIDA score was 115 (range: 81–147); accessibility 55 (range: 46–61), reliability 22 (range: 8–45) and usability 38.5 (range: 21–50), while the median DISCERN score was 43.5 (range: 16–69). The DISCERN score was significantly associated with the overall LIDA score and usability and reliability components of the LIDA score (*p* < 0.001). However, no significant associations were observed between readability and accessibility. A significant correlation was noted between usability and reliability components of the LIDA score (Spearman’s rho: 0.789, p < 0.001).

**Conclusion:**

In this study, the readability, reliability and quality scores of most websites were found to be suboptimal and hence, there is potential for improvement. As the internet is expanding rapidly as a readily available source of information to the public, it is essential to implement steps to ensure that highest quality information is provided without any commercial motivation or bias.

## Background

Testicular cancer mostly affects young men, and it peaks around 20–35 years [1]. American cancer society estimates that 8720 cases of testicular cancer will be diagnosed, and 380 men will die of testicular cancer in the United States of America (USA) during 2016 [2]. Testicular cancer has one of the highest cure rates of all cancers with an overall 5-year survival rate of approximately 95%, although the 5-year survival rate drops to approximately 75% in advanced disease [3]. Adequate knowledge and education about this condition is important among the general public which would facilitate early presentation to healthcare services. Furthermore, it may help diagnosed patients to improve their knowledge which may help them to understand the disease and better navigate the cancer care pathway.

Currently, around half of the world population have access to the internet [4]. With the increasing use of the internet, many patients tend to visit websites to clarify their health-related issues, even before consulting a doctor. In a study conducted in the USA, 87% of the adult population used internet on a regular basis, and of this 72% had checked health related information on the internet within the past year [5]. With the widespread use of internet as an information source, accuracy and reliability of information have become major concerns, especially as there are no regulations or standardizations to ensure accuracy and reliability of information. There are several studies which have shown that information available for patients on the internet on many situations were inaccurate and of inferior quality [6, 7]. Such inaccurate and inferior quality information has the potential to cause serious harm in terms of cancer outcomes and psychological wellbeing.

Based on available data, more than 80% of patients believe that health related information found on the internet is reliable [7]. Further, health information on the internet has been shown to influence perception about the medical condition in addition to its effect on patients’ decision on treatment choices [8].

As many of the internet users are lay people with an average education level, presentation of information for such an audience needs to be simple for them to grasp the full meaning. The National Institute of Health recommends that patient information materials should be written at or below sixth-grade level to ensure that the majority of the users can understand such material [9]. Yet, information available on medical conditions targeting lay audiences have repeatedly been shown to contain information that is far too complex for an average audience to comprehend [10].

This study was conducted with the aim of assessing the quality and readability of patient education information on testicular cancer available on the internet.

## Methods

### Data sources

We searched the search engines Google, Yahoo and Bing which were the top three search engines on the internet. These engines were selected as patients prefer to use general search tools rather than medical search tools, such as PubMed [11, 12]. The initial search was conducted in November 2016 and the searches were repeated again in March 2017 and January/February 2018 to look for updated websites. We searched the web in English language using key words ‘testicular cancer’, ‘testicular tumour’, ‘testicular tumor’, ‘testicular malignancy’, ‘germ cell tumour’ and ‘germ cell tumor’ (without using quotation marks). All searches were done using incognito mode to prevent bias arising from previous searches.

Each keyword received millions of results from each search engine (average approximately 3.3 million per key word per search engine) (Table 1). We kept the default settings and avoided any plug-ins or advanced search options.

**Table 1:** Search result by each search engines

	Testicular cancer	Testicular malignancy	Testicular tumour	Testicular tumor	Germ cell tumour	Germ cell tumor
Google	2,320,000	458,000	1,750,000	1,500,000	787,000	1,440,000
Yahoo	3,960,000	5,020,000	4,890,000	14,000,000	14,800,000	107,000
Bing	4,250,000	324,000	2,500,000	476,000	278,000	888,000

Only the top 50 search results for each search engine for each keyword were analysed as most internet users do not access websites beyond the first 50 results [13]. Of those, only the websites on patient education were selected for analysis, after excluding duplicate websites. Websites that had free access, without a password requirement, presented in English language and provided patient information on testicular cancer were included. Advertisements, and discussion forums were excluded as we felt that those may contain biased information. As this study was about patient-orientated information, sites including resources for clinicians, guidelines and professional articles were also excluded. Sites that fulfilled the inclusion criteria were then assessed by two investigators independently and a common agreement was reached. The readability, accessibility, usability, reliability and quality were assessed by validated instruments. We also assessed the shortcomings of the information provided by the websites in relation to each major aspects of testicular cancer, which includes clinical details, investigations, treatment, quality of life and support groups.

### Readability scores

Readability was defined as the ease of reading and understanding the text. We chose the most commonly used readability score, the Flesch reading ease score (FRES) [14]. The FRES rates text on a 100-point scale based on the average number of syllables per word and words per sentence. A higher score indicates that the material is easier to read. Materials with scores of 90–100 are easily understood by an average 11-year-old. Scores between 60 to 70 are considered as standard readability level which is easily comprehend by 13–15 year-olds [14].

### LIDA tool

LIDA is an online validated tool used to assess the content and design of health information on the web. It has a total of 41 questions where each question is rated on a scale ranging from 0 to 3. The three main domains in LIDA tool are accessibility, usability and reliability of the information [15, 16]. The contents in the scoring system includes clarity, the structural lay out, consistency, ease of navigation, effectiveness of the search facilities, frequency of content update, reliability of the author and reference material.

Accessibility is defined as the availability of material without restrictions while usability is defined as the ease of extracting necessary information from a website. Reliability is defined as being able to obtain accurate and relevant information on the internet. The LIDA scores were considered to be ‘high’ if they were above 90%, ‘moderate’ if they were 50–90% and ‘low’ if they were below 50% [15].

### DISCERN instrument

DISCERN is a questionnaire with 16 questions which are used to assess the quality of the published material. The first eight questions address reliability; questions 9–15 focus on the treatment information on the website and the 16th question assess the overall quality of the website. The contents in the scoring system include source of information, sources of additional support, frequency of content update, quality of information of treatment with benefits and side effects, and information on quality of life. Each question is rated on a 5-point scale ranging from 1 to 5 with a maximum score of 80. A score of 5 indicates high-quality information with minimal shortcomings and a reliable source of information mainly for treatment options. A score of 1 indicates the material has extensive shortcomings and not a good source of information for available treatment options. Hints are provided for each question to help the users to consider the important factors while answering. Ideal patient material by DISCERN will include current levels of evidence, will have multiple treatment options, and provides sources of additional information for further queries [17–19].

### Website certification

There are several certification websites developed to standardize reliability and credibility of information over the internet. We selected the HON code (Health on the Net foundation code of conduct) which is the most widely accepted certification online site. HON which was established in 1995 is an internationally recognized non-profit organization. Currently, more than 7000 websites based in 102 countries use HON code to certify their sites [20]. Furthermore, we categorised websites as governmental and non-governmental. We defined government sites as those maintained by the public health sector of the country. Those that are managed by non-governmental organisations, private institutions or voluntary cancer societies which are not under the direct control of the government sector were taken as non-governmental.

Data were analysed using SPSS software version 17 and were expressed in terms of frequency and percentages. Statistical analysis was done using the Chi-square test and non-parametric tests (Mann Whitney-U test). A *p* value < 0.05 was considered as statistically significant.

## Results

The search using the above keywords resulted in thousands of websites (Table 1). Of them, the first 900 websites (top 50 links from each search engine and key words) were evaluated. There were considerable overlaps and 683 were excluded for duplication of websites. Furthermore, 92 professional websites and 63 advertisements, and discussion fora were excluded. Sixty-two patient-oriented websites were suitable and were included for analysis. The majority (*n* = 57, 91.9%) of these sites were operated by non-governmental organizations.

The relevance, accuracy and the depth of information provided by the individual websites were analysed. Only 20 (32.3%) of the websites had given the author of the content and 24 (38.7%) websites have given the last date of update or frequency of update. Reliable data sources such as journal publications, guidelines and text books were mentioned in 36 (58.1%) websites. All websites had given a correct account on the symptoms of testicular cancer. Investigations and treatment options were correctly mentioned in 58 (93.5%). Benefits and risks of treatment were correctly given in 18 (29%) and 26 (41.9%) websites, respectively. An account of the surgical procedure was mentioned in 45 (72.6%). Details of adjuvant treatment was given in 48 (77.4%). The effects on the quality of life due to testicular cancer was mentioned in 26 (41.9%) websites while details regarding external support groups for testicular cancer patients were mentioned in 33 (53.2%).

The median FRES score of included websites was 51.6 (range 28.1–74.1). Only 11 (17.7%) websites scored 60 or more which is considered as the standard readability level.

The overall median LIDA score was 115 (range 81–147). Scores for the three domains of the LIDA were 55 for accessibility (range 46–61), 22 for reliability (range 8–45) and 38.5 for usability (range 21–50). All websites scored high (37.1%, *n* = 23) or moderate (62.9%, *n* = 39) with regard to accessibility. The majority had low reliability (62.9%, *n* = 39) and the rest had moderate reliability (37.1%, *n* = 23). The majority had moderate (85.5%, *n* = 53) or high (1.6%, *n* = 1) usability and only 12.9% (*n* = 8) had low usability scores.

The median DISCERN score of the websites was 43.5 (range 16–69). A considerable proportion (40.3%, *n* = 25) scored 50% or less (low score) of the total score.

Of the 62 websites, 22 (35.5%) were certified by HON code, of which three appeared in the top 10 based on the DISCERN score.

The analysis between DISCERN score and other parameters is shown in Table 2. Significant association was noted concerning usability, reliability and overall LIDA score (*p* < 0.001). However, there was no significant association with readability and accessibility (Table 2). Furthermore, significant correlation was noted between usability and reliability (Spearman’s rho: 0.789, *p* < 0.001).

**Table 2:** Associations between DISCERN score and other parameters

		DISCERN score				
		Low (*n* = 25)		Moderate/High (*n* = 37)		*p* value
		Median	Range	Median	Range	
Readability score		50	(29.1–67.6)	52	(28.1–74.1)	0.374
LIDA overall score		103	(81–141)	121	(82–147)	< 0.001
Accessibility		56	(48–61)	54	(46–61)	0.569
Reliability		17	(8–34)	26	(9–45)	< 0.001
Usability		32	(21–46)	41	(25–50)	< 0.001
		Number	%	Number	%	
Government	No	24	(96.0%)	33	(89.2%)	0.334
	Yes	1	(4.0%)	4	(10.8%)	
HON certified	No	18	(72.0%)	22	(59.5%)	0.311
	Yes	7	(28.0%)	15	(40.5%)	

The median DISCERN score for the top 10 websites was 60 (range 52–69) while the median readability score was 58.30 (range 42–74). For those sites, median accessibility score was 57 (range 53–59), median usability score was 42 (range 34–50) and median reliability score was 27.5 (range 19–49). Of the top 10 websites related to testicular cancer information, only three websites were certified by HON code (Table 3). Those websites give accurate information covering all major aspects of testicular cancer including symptoms, signs, investigations, treatment types with risks and benefits of each modality of treatment and information on support groups.

**Table 3:** The top 10 websites according to analysis by the DISCERN instrument for testicular cancer

	Website	Organization	Type of organization	Authors	Content update	Data sources	HON Certified website	Non-Governmental website
1	http://www.macmillan.org.uk/information-and-support/testicular-cancer	Macmillan Cancer support	Charity Organization	NA	NA	NA	No	Yes
2	http://www.nhs.uk/conditions/Cancer-of-the-testicle	National Health Service UK	Professional body	NA	Every 3 years	NA	No	No
3	http://www.cancerresearchuk.org/about-cancer/type/testicular-cancer/	Cancer research UK	Charity organization	NA	Annually	Text books, UK Cancer Statistics	No	Yes
4	http://www.cancer.org/cancer/testicularcancer/index	American Cancer society	Charity organization	NA	Last update: 2 years back	NA	Yes	Yes
5	https://www.nlm.nih.gov/medlineplus/ency/article/001288.htm	US National library of Medicine	Professional body	Subject Expert	Last update: 2 years back	Journal articles, National Guidelines	Yes	No
6	http://www.cancer.net/cancer-types/testicular-cancer	American Society of Clinical Oncology	Professional body	Expert editorial board	Last update: 18 months back	NA	Yes	Yes
7	http://www.cancercouncil.com.au/testicular-cancer/diagnosis/	Cancer Council NSW	Charity organization	Expert editorial board	Every 2 years	NA	No	Yes
8	https://www.cancer.gov/types/testicular	National Cancer Institute USA	Government	Expert editorial board	Last update: 18 months back	NA	No	No
9	http://www.oncolink.org/types/article.cfm?c=19&id=9459	OncoLink, USA	Charity organization	Expert editorial board	Last edited: 1 year ago	Journal articles, guidelines	No	Yes
10	https://www.emedicinehealth.com/cancer_of_the_testicle/article_em.htm#testicular_cancer_facts	eMedicinehealth	Professional body	Expert editorial board	Last edited: 3 months back	Journal articles	No	Yes

(*NA* information not available on the website)

## Discussion

Our study findings indicate that patient education material related to testicular malignancy are relatively easy to access on the internet. However, a considerable proportion of the websites were not adequately relevant as patient education material. Therefore, the readers are forced to browse through a large number of sites to find out the relevant websites which may be time-consuming, and many users may not be able to perform this adequately. Even after accessing the sites with relevant information, many users may not be able to comprehend the information as the readability scores of a majority of the websites were found to be high for an average lay audience. For instance, the median FRES score of the websites was 51.6, which is equal to 10th to 12th-grade readability level that is categorized as difficult to read. Further, only 11 (17.7%) websites scored 60 or more which is the standard readability level.

Based on the LIDA tool, we found that most of the websites belonged to ‘moderate’ category in relation to accessibility and usability. However, the majority had low reliability (63%) which needs to be addressed seriously. Regular assessment regarding the reliability of the content of the websites by experts in the field may be required to maintain acceptable standards.

Analysis by the DISCERN instrument revealed that testicular cancer websites in general have shortcomings in quality and reliability of information related to treatment options. Although the overall median score for DISCERN was low at 43.5 and a considerable proportion (40%) scored less than 50% of the total score, the top 10 websites provided relatively better-quality with a median DISCERN score of 60 (75% of the total score).

Each search engines produce search results using different methods. The exact details related to the search engines’ mechanisms are commercial secrets, particularly regarding results ranking. Therefore, their mode of operation is only broadly known. In terms of the results delivery and prioritising, there are three key operations: crawling, results matching, and results ranking. Due to the variations in the algorithm used by the search engines the results may vary depending on the search engine used. This explains the vast variations seen in Table 1. Furthermore, search engine algorithms are updated frequently and thus the search results and the popularity of individual results and the ranking change with time [21].

There were several important shortcomings related to relevance and reliability. Only 32% of the websites have given the author of the content, although the content was accurate. Mentioning the author is likely to increase the credibility of the article and will also influence the responsibility of the author. Furthermore, reliable data sources such as journal publications, guidelines and textbooks which increase the perceived reliability of a website was mentioned only in 58% of websites. Although the websites mention the details of testicular cancer accurately, the information was found to be incomplete. For example, the benefits and risks of treatment were correctly given only in 29% and 42%, respectively. Similarly, the effects on the quality of life and details regarding external support groups was mentioned in 42% and 53% respectively. An ideal website should cover all aspects including quality of life and support groups in simple language.

Although LIDA and DISCERN give an overall assessment on the reliability, it does not assess the accuracy of the individual components such as clinical features, tests, quality of life etc. Furthermore, they do not provide assessment regarding the accuracy of information specific to testicular cancer. However, they do include the frequency of content updating and reliability of the content and the credibility of the author/source of information to the scoring system.

It is certain that more and more patients will continue to use online websites to get information. Thus, measures should be taken to ensure that the websites and reliable, accurate, complete, well-maintained and under the direct control of trusted well known official medical authorities. Reputed medical journals have started to include patient education articles such as the recently published article on testicular mass [22]. However, further steps would be needed to improve completeness. Until such time, as a short-term measure, all patient education websites could display the scores related to quality, reliability and readability on every web page. In the longer term, measures will have to be developed to certify websites based on their scoring. Further, we suggest that national cancer services develop good quality, reliable and easily readable information related to the diagnosis and treatment of testicular cancer. Special emphasis should be given to the section on treatment modalities available as there were shortcomings observed in this study related to treatment options. Physicians also have an obligation to be aware of the information available on the internet and websites that contain reliable information in relation to their speciality. Hence, a knowledge on the scoring systems and scores of different websites in addition to their individual assessment of the websites, may help physicians better understand and identify websites suited for their patients. This would allow the physicians to recommend websites that contain good quality and reliable information which may cater to different patient needs.

There are several limitations in this study. We used the most popular search engines in our study. The default settings may be different according to the geographical location and time. If different settings were used, the search results may vary. There are shortcomings in assessing readability using readability formulae. Short sentences in simple words will give a higher FRES score which may not be interesting to the reader. They do not assess the organization and lay out of a web page or use of prompt diagrams. Therefore, these instruments can only be used as an aid for the assessment of readability. The LIDA tool and DISCERN instrument comprise of many criteria to evaluated websites related to health. However, they are subjective assessments and may have given rise to some minor discrepancies.

## Conclusion

In this study, the readability, accessibility, usability and quality scores of most websites were found to be moderate and the reliability of websites were found to be considerably low. Therefore, there is a need and potential for major improvements. Access to reliable information will help promote early presentation and will also aid in informed decisions on treatment. Development of certification systems for patient education websites which would help patients identify good quality information resources and promoting national cancer services to develop good quality patient information web sites that suits the local setup are identified as potential methods to overcome the observed shortcomings. As more and more people access internet for health-related information, such measures may help improve the quality of these websites and thereby patient understanding of the disease which may help improve outcomes.

## Abbreviations

FRES: Flesch reading ease score
HON: Health of the net
USA: United States of America
